# The Conserved Coronavirus Macrodomain Promotes Virulence and Suppresses the Innate Immune Response during Severe Acute Respiratory Syndrome Coronavirus Infection

**DOI:** 10.1128/mBio.01721-16

**Published:** 2016-12-13

**Authors:** Anthony R. Fehr, Rudragouda Channappanavar, Gytis Jankevicius, Craig Fett, Jincun Zhao, Jeremiah Athmer, David K. Meyerholz, Ivan Ahel, Stanley Perlman

**Affiliations:** a Department of Microbiology, University of Iowa, Iowa City, Iowa, USA; b Department of Pathology, University of Iowa, Iowa City, Iowa, USA; c Sir William Dunn School of Pathology, University of Oxford, Oxford, United Kingdom; d State Key Laboratory of Respiratory Diseases, Guangzhou Institute of Respiratory Disease, The First Affiliated Hospital of Guangzhou Medical University, Yuexiu District, Guangzhou, Guangdong, China

## Abstract

ADP-ribosylation is a common posttranslational modification that may have antiviral properties and impact innate immunity. To regulate this activity, macrodomain proteins enzymatically remove covalently attached ADP-ribose from protein targets. All members of the *Coronavirinae*, a subfamily of positive-sense RNA viruses, contain a highly conserved macrodomain within nonstructural protein 3 (nsp3). However, its function or targets during infection remain unknown. We identified several macrodomain mutations that greatly reduced nsp3’s de-ADP-ribosylation activity *in vitro*. Next, we created recombinant severe acute respiratory syndrome coronavirus (SARS-CoV) strains with these mutations. These mutations led to virus attenuation and a modest reduction of viral loads in infected mice, despite normal replication in cell culture. Further, macrodomain mutant virus elicited an early, enhanced interferon (IFN), interferon-stimulated gene (ISG), and proinflammatory cytokine response in mice and in a human bronchial epithelial cell line. Using a coinfection assay, we found that inclusion of mutant virus in the inoculum protected mice from an otherwise lethal SARS-CoV infection without reducing virus loads, indicating that the changes in innate immune response were physiologically significant. In conclusion, we have established a novel function for the SARS-CoV macrodomain that implicates ADP-ribose in the regulation of the innate immune response and helps to demonstrate why this domain is conserved in CoVs.

## IMPORTANCE

The macrodomain is a ubiquitous structural domain that removes ADP-ribose from proteins, reversing the activity of ADP-ribosyltransferases. All coronaviruses contain a macrodomain, suggesting that ADP-ribosylation impacts coronavirus infection. However, its function during infection remains unknown. Here, we found that the macrodomain is an important virulence factor for a highly pathogenic human CoV, SARS-CoV. Viruses with macrodomain mutations that abrogate its ability to remove ADP-ribose from protein were unable to cause lethal disease in mice. Importantly, the SARS-CoV macrodomain suppressed the innate immune response during infection. Our data suggest that an early innate immune response can protect mice from lethal disease. Understanding the mechanism used by this enzyme to promote disease will open up novel avenues for coronavirus therapies and give further insight into the role of macrodomains in viral pathogenesis.

## INTRODUCTION

Posttranslational modifications (PTMs) of proteins regulate many cellular processes. Common PTMs include phosphorylation, acetylation, ubiquitination, and ADP-ribosylation. Viruses are well known for their ability to manipulate PTMs for their advantage, often encoding proteins that are able to add modifications (protein kinases, E3 ligases) or remove them (phosphatases, deubiquitinases) from proteins. ADP-ribosylation is a common but little-studied PTM whereby diphtheria toxin-like ADP-ribosyl transferases (ARTDs) transfer ADP-ribose from NAD^+^ onto target proteins. ADP-ribose can be attached either as a single unit via mono-ADP-ribosylation (MAR) or as polymers via poly-ADP-ribosylation (PAR). ARTDs have both antiviral and proviral effects on replication (reviewed in reference 1). In support of their antiviral roles, several ARTDs are induced by interferon (IFN) (2, 3); others enhance interferon-stimulated gene (ISG) expression (4, 5); and some are under positive selection, a hallmark of proteins involved in virus-host conflict (6, 7). Specific examples of antiviral ARTD1s include ARTD10, ARTD12, and ARTD14, which block alphavirus replication (2). Also, ARTD14 and ARTD12 have been demonstrated to modulate IFN and NF-κB pathways, respectively (8, 9). Finally, ARTD13, known as zinc-antiviral protein (ZAP), is an inactive ARTD that has strong activity against a number of viruses (10).

Several proteins have been identified that regulate ADP-ribosylation by removing ADP-ribose from target proteins. These include PAR-glycohydrolases (PARG), ADP-ribosyl hydrolases (ARH), and macrodomain-containing proteins (reviewed in reference 11). A macrodomain is an evolutionarily conserved domain consisting of approximately 170 amino acids with a well-defined structure of central β-sheets flanked by α-helices (12, 13). They are present in all domains of life, and humans have 9 genes that encode macrodomain proteins. Many studies have shown that these domains bind to mono- or poly-ADP-ribose (MAR or PAR) and that several are able to hydrolyze ADP-ribose-1′′ phosphate, a by-product of tRNA splicing, to ADP-ribose (14, 15). Recently, these domains were shown to remove ADP-ribose from proteins and thus may play a key role in the regulation of protein ADP-ribosylation (16–18). Furthermore, as several ARTDs have been shown to have antiviral activity, macrodomains may inhibit these functions; however, this has not been experimentally demonstrated. Consistent with this idea, viruses from the *Hepeviridae*, *Togaviridae*, and *Coronaviridae* families all encode macrodomains. Originally, it was thought that these enzymes primarily act as ADP-ribose-1′′-phosphatases (ADRPs) or bind to PAR (14, 19–22), as these activities had been convincingly demonstrated *in vitro*. However, it was recently shown that macrodomains from hepatitis E virus (HEV) and SARS-CoV can deMARylate and dePARylate protein substrates *in vitro* (23), although no specific protein targets for viral macrodomains have been identified. No clear role for any potential macrodomain activity during infection has been identified, and no connection to innate immunity has been shown for ADR-1′′-phosphate or free ADP-ribose.

Coronaviruses (CoVs) are large, positive-sense RNA viruses that cause a variety of veterinary and human diseases. In humans, CoVs were originally thought to cause only mild respiratory or gastrointestinal disease. Then, in 2002 to 2003, severe acute respiratory syndrome (SARS)-CoV emerged, spreading across multiple countries and causing a severe respiratory disease with a mortality rate of ~10% (24). In 2012, the Middle East respiratory syndrome coronavirus (MERS-CoV) emerged as another highly pathogenic CoV with a high mortality rate (25). In addition, a number of SARS-like CoVs are currently circulating in bats and are able to infect human cells, suggesting that a single zoonotic transmission event could set off another epidemic (26). Currently, there are no approved therapeutics or vaccines to treat human CoVs, and as such, increased research into further understanding CoV biology to identify therapeutic targets and novel vaccine strategies is needed to control these viruses.

All known CoVs encode a macrodomain within the large transmembrane protein nonstructural protein 3 (nsp3). Using mutation of the highly conserved asparagine residue, which was shown to be essential for ADP-ribose-1′-phosphatase activity *in vitro* (14), this domain was shown to be required for murine hepatitis virus (MHV) virulence in mice, despite only minor replication defects being associated with mutant viruses *in vitro* (19, 27). In the context of infection with the HKU-39849 strain of SARS-CoV, macrodomain mutation rendered virus highly sensitive to IFN, although this same phenotype was not shown in the MHV studies (19, 20, 27). That study also found increased levels of IFN and CXCL-10 in macrodomain mutant-infected 293 cells (20). The macrodomain has also been analyzed in other viruses. The Sindbis virus macrodomain was required for replication in neuronal cells and virulence in 2-week-old mice (28). Also, while the role of the macrodomain in HEV infection has not been specifically analyzed, it was shown to block interferon production when expressed in isolation (29). Taken together, those studies suggest that the viral macrodomains impact host innate immune pathways to promote disease. While it has been clearly demonstrated that the CoV macrodomain is essential for virulence in MHV, it remains unclear whether this domain is also critical for virulence in pathogenic human respiratory CoV infection. Here we used a mouse-adapted SARS-CoV (MA15) that causes a lethal infection in BALB/c mice (30). We developed a series of recombinant viruses with mutations in the ADP-ribose binding pocket of the macrodomain, which we predicted would reduce or eliminate enzymatic activity, and confirmed this prediction in an *in vitro* deMARylation assay. We found that all of these mutant viruses were highly attenuated and unable to cause lung disease in mice, while *in vitro* replication was unaffected. In addition, we found that the catalytic activity of the macrodomain was required to repress cytokine expression both *in vivo* and *in vitro*. Our results suggest that the SARS-CoV macrodomain functions to suppress the expression of innate immune genes and promote virulence.

## RESULTS

### Creation of SARS-CoV MA15 macrodomain mutants.

**FIG 1:**
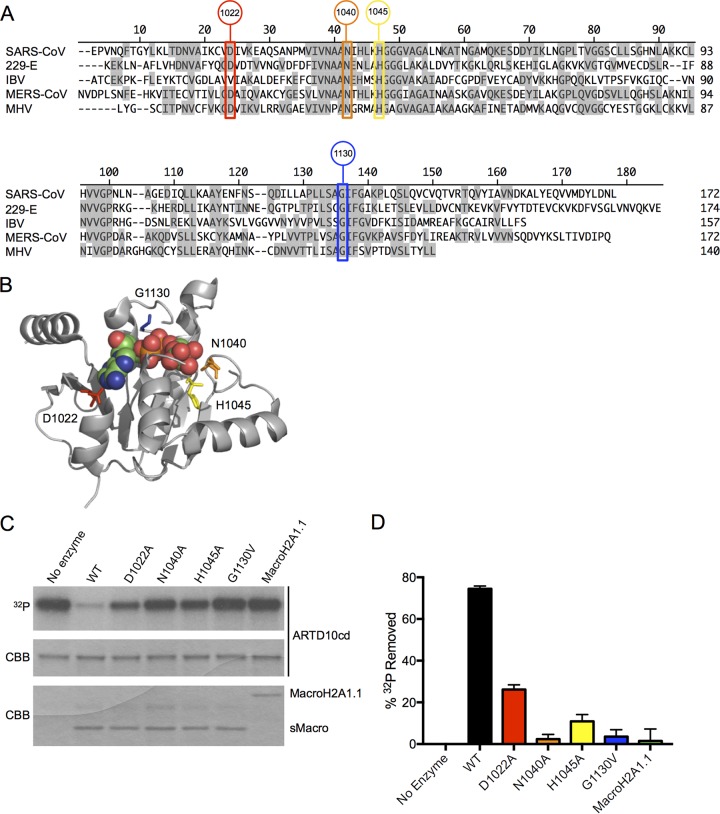
Generation of SARS-CoV macrodomain mutants. (A) The presented alignment was produced using MegAlign of DNASTAR and sequences obtained from NCBI. Amino acids that were mutated in the present study are highlighted. Their amino acid locations in polyprotein 1a are depicted in circles placed above each amino acid. IBV, infectious bronchitis virus. (B) Crystal structure of SARS-CoV macrodomain with mutated amino acids indicated. The crystal structure was obtained from PDB (2FAV) and modified in Pymol. (C) De-ADP-ribosylation of ARTD10 catalytic domain (cd) by SARS-CoV macrodomains as observed by SDS-PAGE followed by autoradiography (^32^P) and Coomassie blue staining (CBB) of ARTD10 and macrodomain proteins. (D) Quantitative analysis of de-ADP-ribosylating activity represented in panel C. Results represent the combined data from 4 independent experiments.

To determine the role of the SARS-CoV macrodomain (sMacro), we identified and then mutated several amino acids predicted to be involved in de-ADP-ribosylating proteins (Fig. 1A and B). The structure of the CoV macrodomain is well defined, and amino acids that line the ADP-ribose binding pocket have been identified (14, 22, 31). Furthermore, several amino acids in this pocket have been tested for ADP-ribose-1′′-phosphatase (ADRP) or O-acetyl-ADP-ribose deacetylase activity with either the SARS-CoV or other related macrodomain proteins (14, 15, 32). As the C1′′ atom of the distal ribose is attacked by ADRP as well as during the process of de-ADP-ribosylation, it is likely that the two types of enzymatic activity utilize the same or similar amino acids (16). Using 2-step Red recombination in concert with either galactose-kinase-mediated or homing endonuclease I-SceI-mediated negative selection (33, 34), we mutated the following amino acids in the SARS-CoV MA15 bacterial artificial chromosome (BAC) (35): D1022A, N1040A, H1045A, and G1130V (Fig. 1A and B). N1040 and H1045 both interact with the terminal ribose through hydrogen bonding with a water molecule that ultimately makes a nucleophilic attack at the C1′′ atom of the terminal ribose (14, 31). Using bacterially expressed recombinant proteins, the H1045A mutation was shown to reduce but not eliminate ADRP activity, while the N1040A mutation completely eliminated ADRP activity (14). Importantly, the N1040 amino acid is universally conserved among enzymatically active macrodomains (15, 16). D1022 has been shown to provide critical hydrogen bonds with water molecules near the adenine ring, stabilizing this interaction (14, 22, 31). Finally, G1130 interacts with the distal phosphate group of ADP-ribose and has been shown to affect macrodomain catalytic activity (15, 32).

The recombinant MA15 BACs were transfected into Vero cells, and the resulting virus was collected, plaque purified, and amplified. Viral sequences were analyzed after 4 passages to determine if the mutations were stable. Real-time quantitative PCR (RT-qPCR) analysis of virus-infected cells followed by direct sequencing verified that all of the mutations were present after at least 4 passages (data not shown). Nsp3 was detected by Western blotting and was found to be in distinct cytoplasmic puncta at 48 h postinfection (hpi) following infection with each virus. None of the mutations had an effect on nsp3 localization, and only small differences in nsp3 protein levels were detected in some of the mutants (see Fig. S1A and B in the supplemental material). Finally, we created bacterially expressed recombinant macrodomain wild-type and mutant proteins using a previously published assay that tested their ability to deMARylate the ADP-ribosylated ARTD10 catalytic domain (ARTD10cd) *in vitro* (23). The macroH2A1.1 protein was utilized as a negative control as it does not contain de-ADP-ribosylating activity (16). The N1040A and G1130V macrodomain mutant proteins were unable to deMARylate ARTD10, while D1022A and H1045A retained a small amount of deMARylating activity compared to wild-type protein (Fig. 1C and D). This demonstrates that these amino acids in the ADP-ribose binding pocket are critical for the SARS-CoV macrodomain to deMARylate protein substrates.

### SARS-CoV MA15 macrodomain mutants are not required for replication or IFN resistance in tissue culture.

**FIG 2:**
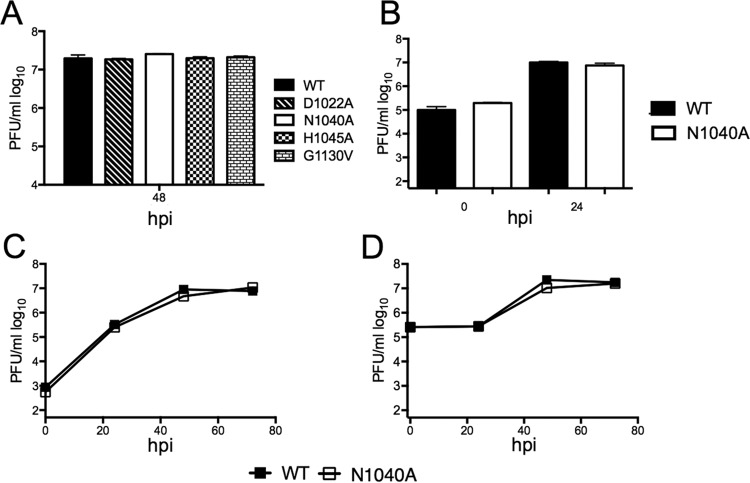
SARS-CoV macrodomain enzymatic activity is not required for replication *in vitro*. (A to C) Vero cells were infected with the indicated virus at an MOI of 0.01 (A), 1 (B), or 0.001 (C) PFU/cell. Progeny virus was collected at the indicated time points, and yields were determined by plaque assay. (D) Calu-3 2B4 cells (a kind gift from Kent Tseng) were infected with wild-type (WT) or N1040A virus at an MOI of 0.5 PFU/cell. Progeny virus from supernatant was collected at the indicated time points, and yields were determined by plaque assay.

Previously published results demonstrated that CoV macrodomain mutants have little to no effect on replication *in vitro* (19–21, 27). Consistent with these observations, all of the MA15 macrodomain mutants described above reached nearly identical peak titers at 48 hpi following inoculation with a low multiplicity of infection (MOI) of Vero E6 cells (Fig. 2A). A more detailed analysis of growth kinetics was carried out for the N1040A mutation, as we used this virus extensively. We found no significant differences in virus growth after inoculation of Vero or the Calu-3 2B4 human bronchial epithelial cell line cells at a low or high MOI (Fig. 2B to D).

It was previously shown by Kuri et al. that the SARS-CoV HKU-39849 strain N1040A mutant virus was more sensitive to IFN than wild-type virus *in vitro* (20). In contrast, the corresponding mutants in MHV did not have increased sensitivity to IFN (19, 27). To assess the IFN sensitivity of the MA15 N1040A virus, Vero E6 cells were pretreated with increasing amounts of alpha IFN (IFN-α) or IFN-β and then infected with the MA15 wild-type strain and MA15-N1040A. IFN was retained on the cells for the duration of the experiment, and the cells and supernatants were analyzed at 48 hpi for viral titers and genomic RNA (gRNA) (Fig. S2A to C). Titers and gRNA levels for both viruses were reduced by about 2-logs following addition of 100 units of IFN-β (Fig. S2B and C), but neither virus was affected by up to 1,000 units of IFN-α (Fig. S2A). The differences in sensitivity to IFN-β and IFN-α were consistent with previous reports (36). The differences in IFN sensitivity of N1040A virus between this study and that of Kuri et al. could be due to a variety of factors, including virus strain, types of IFN used, or second-site mutations that were not controlled for in the previous study (20). We conclude that the catalytic activity of the SARS-CoV MA15 macrodomain is nonessential for virus replication or IFN resistance *in vitro*.

### Macrodomain mutant viruses are attenuated in a mouse model of severe respiratory disease.

**FIG 3:**
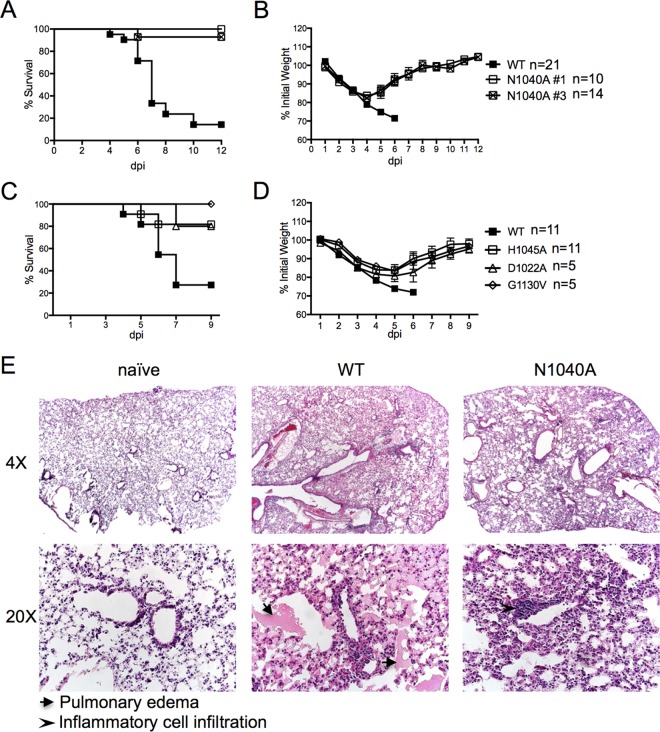
SARS-CoV macrodomain enzymatic activity promotes lung pathology during a lethal SARS-CoV infection. Young 7- to 9-week-old female BALB/c mice were intranasally (i.n.) infected with 3 × 10^4^ PFU of recombinant SARS-CoV MA15. N1040A #1 and #3 were derived from two different bacterial colonies. Survival (A and C) and percent weight loss (B and D) were measured for 10 to 12 days. (A and B) WT, *n* = 21 mice; N1040A #1, *n* = 10 mice; N1040A #3, *n* = 14 mice. (C and D) WT, *n* = 11 mice; H1045A, *n* = 11 mice; D1022A, 5 mice; G1130V, 5 mice. (E) Histological analysis of lungs from WT- and N1040A-infected mice at 5 dpi. Examples of pulmonary edema, found in WT-infected lungs, are marked with arrowheads. *n* = 3 mice per group.

To test whether the macrodomain is required for SARS-CoV virulence, we infected female BALB/c mice and monitored them for weight loss and survival. Whereas approximately 80% of mice infected with wild-type virus succumbed to infection, >80% of mice infected with the mutant viruses survived, although all lost weight (Fig. 3A to D). Interestingly, nearly 100% of mice survived infection with mutant viruses completely devoid of deMARylating activity (N1040A and G1130V), while slightly fewer mice survived infection with mutant viruses that retained some deMARylating activity (D1022A and H1045A) (Fig. 1C; see also Fig. 3A and C). This suggests that virulence correlates with enzymatic activity. Histological analysis of the lungs at 5 days postinfection (dpi) found that wild-type-infected mice had large amounts of pulmonary edema in the lungs, while those of N1040A mutant virus-infected mice were devoid of edema (Fig. 3E). Since the disease phenotypes were similar in mice infected with all the mutant viruses, we used the N1040A mutant exclusively for all subsequent experiments.

**FIG 4:**
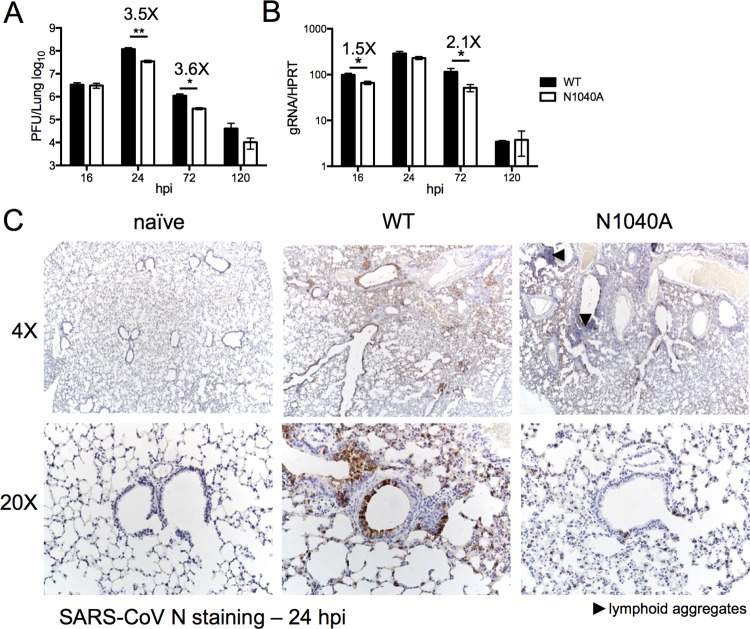
N1040A virus infection results in reduced viral loads *in vivo*. (A and B) BALB/c mice were infected as described above, and lung titers (A) and gRNA levels (B) were determined by plaque assay and RT-qPCR with primers specific for nsp12 and normalized to HPRT, respectively. *n* = 4 to 8 mice per group for panels A and B. (C) Immunohistochemical examination of SARS-CoV N protein at 24 hpi in both WT and N1040A virus-infected lungs. N1040A-infected lungs appeared to exhibit less antigen staining. *n* = 3 mice per group. *, *P* ≤ 0.05; **, *P* ≤ 0.01; ***, *P* ≤ 0.001.

To determine if the attenuation of N1040A virus correlated with a reduced virus load, we measured viral titers in the lungs of infected mice. While viral loads were initially similar at 16 h postinfection (hpi), there were approximately 3- to 4-fold reductions in virus titers following N1040A infection at 24 and 72 hpi, as well as significant differences in levels of viral genomic RNA (gRNA) during infection (Fig. 4A and B). Previous studies have found extensive viral N protein staining around the airways and in the parenchyma with extensive N protein distribution in type II and, to a lesser extent, type I pneumocytes at 24 hpi (37, 38). Viral N protein was similarly distributed in lungs infected with wild-type or N1040A virus, but it appeared that less N protein was detected in N1040A-infected samples, consistent with the titer data (Fig. 4C). These data demonstrate that the SARS-CoV macrodomain modestly affects virus replication *in vivo* and is important for SARS-CoV-mediated lethality in infected mice.

### The SARS-CoV macrodomain suppresses IFN, ISG, and proinflammatory cytokine expression in mice during the early stages of infection.

**FIG 5:**
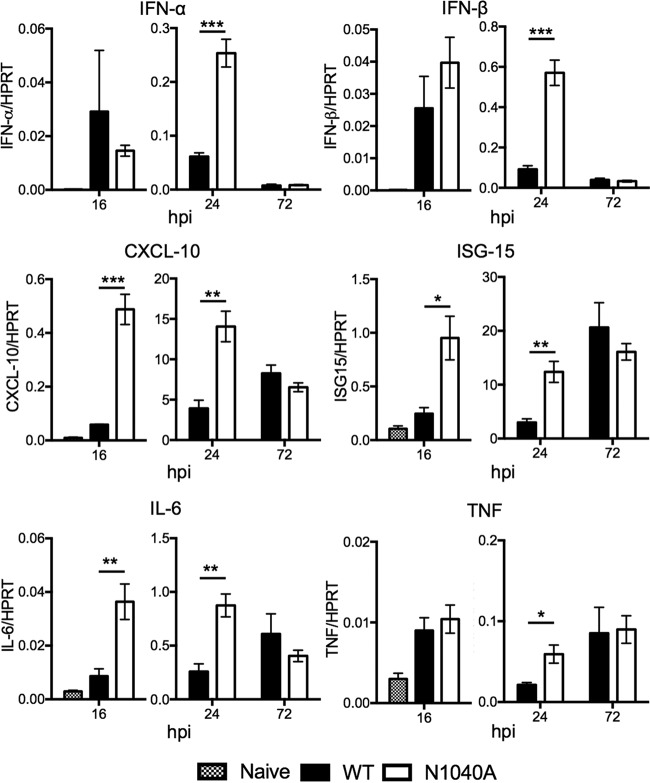
SARS-CoV macrodomain enzymatic activity suppresses early cytokine production *in vivo*. BALB/c mice were infected as described above, lungs were harvested at the indicated times, and transcript levels were determined by RT-qPCR with primers specific for each transcript and normalized to HPRT. *n* = 4 mice per group/experiment. Data are derived from results from a single experiment, representative of 2 to 3 independent experiments.

To begin to address the mechanism of virus attenuation, we analyzed the expression of IFN and other cytokines in the lungs during infection. We found a significant increase in levels of IFN-α and IFN-β, interferon-stimulated genes (ISGs) CXCL-10 and ISG15, and the proinflammatory cytokines interleukin-6 (IL-6) and tumor necrosis factor (TNF) at 16 to 24 hpi following infection with N1040A (Fig. 5). Since N1040A infection results in a reduced viral load at 24 hpi that is concurrent with this increase in IFN production, we hypothesized that IFN may be responsible for reducing the viral load. To test this, we measured lung virus titers at 24 hpi in the absence of IFN-I signaling (IFNAR^−/−^ mice). Surprisingly, infection of IFNAR^−/−^ mice with N1040A virus still resulted in an ~3-fold reduction in viral load at 24 hpi compared to infection with wild-type virus (Fig. S3). The reduction in virus load could be due to a cytokine that does not use the type I IFN receptor, such as IFN-λ. Alternatively, it may suggest that the macrodomain promotes virus replication *in vivo* and suppresses cytokine expression independently or that it targets a protein that can separately affect the two processes.

### SARS-CoV N1040A infection does not affect the accumulation of innate immune cells into the lung.

Next, to assess whether the increased cytokine response correlated with differences in inflammatory cell accumulation, we measured frequencies and cell numbers at 1 and 3 days postinfection (dpi), since wild-type-infected mice succumb to infection beginning at day 4. While the frequencies of neutrophils and monocytes/macrophages were reduced at day 3 after N1040A virus infection, there were no significant differences in the numbers of any innate immune cell type, except for a slight increase in the numbers of dendritic cells at day 3 (Fig. S4). There were small but significant increases in the amount of CD4 and CD8 T cells in the lung at these times, possibly reflecting increased expression of CXCL-10 and CCL2 in N1040A-infected mice (Fig. S4) (39, 40). However, at those early time points it is unlikely that these were virus-specific T cells, making their significance unclear. These results suggest that the enzymatic function of the macrodomain has minimal effects on innate immune cell accumulation during the early stages of infection.

### The SARS-CoV macrodomain represses innate immune gene expression *in vitro*.

**FIG 6:**
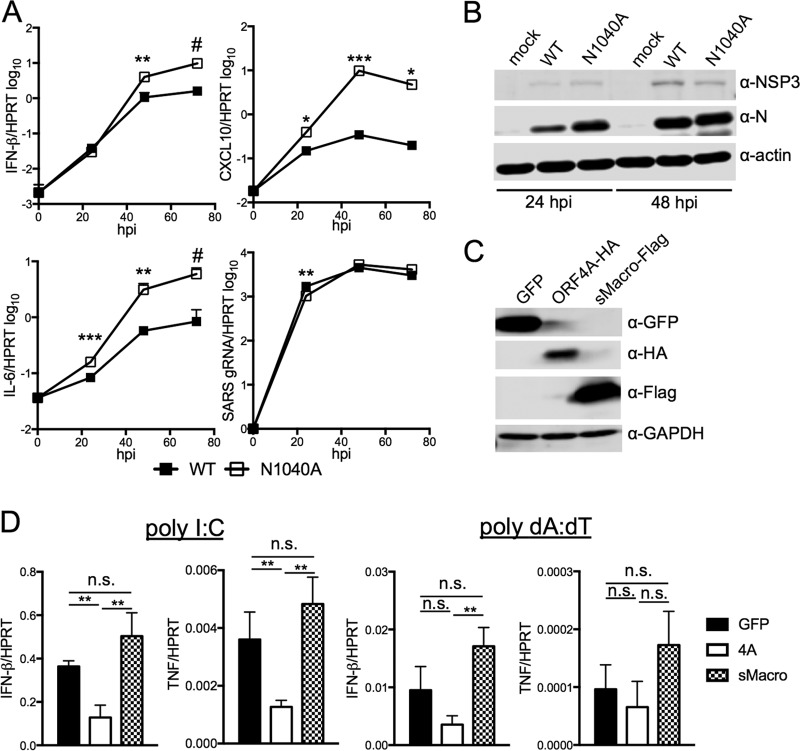
The SARS-CoV macrodomain is required but not sufficient to suppress cytokine expression *in vitro*. (A) Calu-3 2B4 cells were infected with WT or N1040A virus at an MOI of 2 PFU/cell. Cells were collected at the indicated time points, and RNA levels were determined by RT-qPCR with primers specific for each transcript and normalized to HPRT. Data are derived from results of 1 experiment representative of 3 independent experiments. (B) Calu-3 2B4 cells were infected as described for panel A, and cells were collected at 48 hpi. Lysates were analyzed by immunoblotting with the indicated antibodies using a Li-COR Odyssey Imager. (C and D) pcDNA3-GFP, pLKO-MERS-CoV ORF4A, and pcDNA3-sMacro were transfected into HeLa cells. At 24 h later, the cells were either collected for immunoblotting (C) or transfected with 1 μg pI-C or 1 μg dA-dT (D). For panel D, cells were collected at 16 h after transfection and RNA was analyzed by RT-qPCR with primers specific for indicated transcript. HA, hemagglutinin; GAPDH, glyceraldehyde-3-phosphate dehydrogenase. Data are derived from results of 1 experiment representative of 2 independent experiments. *, *P* ≤ 0.05; **, *P* ≤ 0.01; ***, *P* ≤ 0.001; #, *P* = 0.06; n.s., not significant.

As Sars-CoV primarily infects airway and alveolar epithelial cells, we next tested whether the macrodomain could antagonize innate immune signaling from infected epithelial cells *in vitro*. In Calu-3 2B4 cells, a bronchial epithelial cell line (41), wild-type virus induced only a small increase in innate immune gene expression, while expression was dramatically elevated following N1040A virus infection. Four-, 30-, and 5-fold increases in IFN-β, CXCL-10, and IL-6 levels were observed at 48 hpi, respectively, even though no differences in genomic RNA or N protein accumulation levels were detected (Fig. 6A and B).

While these experiments clearly demonstrate that the SARS-CoV macrodomain suppresses innate immune gene expression during infection, whether this suppression occurs in isolation is not known. We expressed the SARS-CoV macrodomain (sMacro) using the same amino acid sequence as that of the wild-type protein used for the deMARylating assay (Fig. 1C) and, as a positive control, the MERS-4A protein in HeLa cells (Fig. 6C). We then tested whether this domain could block poly(I-C)- or poly(dA-dT)-induced expression of IFN-β or TNF-α (42). Poly(I-C) induces interferon by activating MDA5, while poly(dA-dT) activates RIG-I. Poly(I-C) induced IFN and TNF in control green fluorescent protein (GFP)-expressing cells 600- and 200-fold, while poly(dA-dT) induced IFN and TNF 16- and 6-fold, respectively. The sMacro protein was unable to block induction of IFN-β or TNF by either method, while MERS-4a reduced these transcripts by ~3-fold, except in the case of poly(dA-dT)-induced TNF, where it had no effect (Fig. 6D). We conclude that the SARS-CoV macrodomain alone is not able to suppress innate immune gene expression.

### Coinfection with wild-type and N1040A virus enhances survival of SARS-CoV MA15-infected mice.

**FIG 7:**
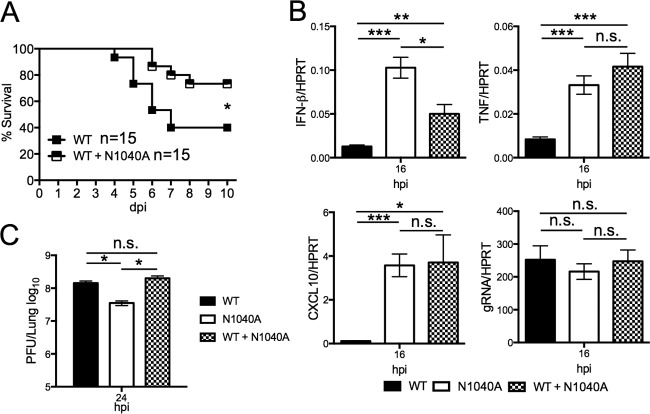
Mice coinfected with WT and N1040A virus have better outcomes than mice infected with WT virus alone. (A) BALB/c mice were infected with 3 × 10^4^ PFU of WT virus alone or coinfected with 3 × 10^4^ PFU of both WT and N1040A virus. Survival was monitored for 10 days. *n* = 15 mice for each group. (B) BALB/c mice were infected with 3 × 10^4^ PFU of WT virus or N1040A virus or coinfected with 3 × 10^4^ PFU of both WT and N1040A as described, lungs were harvested at the indicated times, and indicated transcript levels were determined by RT-qPCR with primers specific for each individual gene and then were normalized to HPRT. *n* = 4 mice per group. (C) BALB/c mice were infected as described for panel B, and virus and lung titers were determined by plaque assay. *n* = 3 to 4 mice per group. Data are derived from results of 1 experiment representative of 2 independent experiments. *, *P* ≤ 0.05; **, *P* ≤ 0.01; ***, *P* ≤ 0.001; n.s., not significant.

To separate the effects of virus replication from cytokine production in the attenuation of N1040A virus, we compared the viral loads, innate immune gene expression levels, and survival rates of wild-type-infected mice and mice coinfected with wild-type and N1040A virus. At the dose of 3 × 10^4^ PFU of each virus (total, 6 × 10^4^ PFU), fewer coinfected mice succumbed to infection than mice infected with only wild-type virus at a dose of 3 × 10^4^ PFU (Fig. 7A). These dually infected mice also had increased IFN and proinflammatory cytokine levels at 16 hpi that were comparable to the levels seen with N1040A-infected mice but titers identical to those seen with mice infected with the wild-type virus at 24 hpi (Fig. 7B and C). This suggests that an early innate immune response during coinfection protects mice without affecting virus replication. The protection was incomplete, as ~25% of mice did succumb to the coinfection compared to only ~5% after infection with N1040A virus only (Fig. 3A and 7A). This result suggests that the early innate immune response during MA-15 N1040A infection was at least partially responsible for its attenuation.

## DISCUSSION

Results presented here demonstrate that the SARS-CoV nsp3 macrodomain is critical for virulence. It was also required for optimal virus replication *in vivo* and cytokine repression both *in vivo* and *in vitro*. These results are consistent with reports demonstrating an important *in vivo* role for CoV macrodomains. Specifically, catalytic mutants with mutations of the macrodomain in murine MHV and in Sindbis virus were unable to cause severe hepatitis and encephalitis, respectively (19, 27, 28). However, in addition to demonstrating reduced replication *in vivo*, our results indicate a role for the conserved SARS-CoV macrodomain in suppressing the early IFN and proinflammatory cytokine response and promoting pathological changes, such as edema, in the lung and lethality in infected mice. Specifically, SARS-CoV N1040A, a virus devoid of macrodomain catalytic activity, induced significantly elevated expression levels of IFN, ISGs, and other cytokines at 16 to 24 hpi. Importantly, mice coinfected with both wild-type and N1040A had better outcomes and increased IFN and proinflammatory cytokine expression than mice infected with wild-type virus, despite the presence of similar virus titers at 24 hpi. This suggests that an early innate immune response plays an important role in protecting mice from lethal disease. However, dually infected mice were not completely protected from infection, suggesting that the ability of the macrodomain to augment virus load *in vivo* also plays a significant role in its ability to promote disease.

It has long been known that SARS-CoV is able to repress the cellular IFN response, and a number of SARS-CoV proteins have been shown to block the IFN response in isolation or *in vitro* (43–47). This report presents the first example of a SARS-CoV mutant virus whose activity leads to an elevated level of antiviral cytokines *in vivo* and *in vitro*, thus clearly demonstrating a prominent role for the macrodomain in inhibiting the early innate immune response.

Notably, we were unable to definitively address the role of IFN-I in protection using IFNAR^−/−^ mice, due to IFN having dual roles during infection. We recently showed that a dysregulated IFN response enhanced virulence of SARS-CoV (37). SARS-CoV strongly represses IFN production initially following infection, and mice are completely protected from disease if exogenous IFN is given before peak replication, demonstrating an important role for early IFN-I production in protection. In the absence of exogenous IFN, IFN-I is rapidly produced at later times and recruits inflammatory monocytes to the lung. There, these monocytes produce additional proinflammatory cytokines, cause vascular leakage, and impair virus specific T-cell responses, ultimately leading to lethality (37). Consequently, SARS-CoV is unable to cause disease in IFNAR^−/−^ mice. It is currently unknown how an early IFN/cytokine response protects mice from SARS-CoV-mediated disease. Identifying the precise mechanism of cytokine-mediated protection from a lethal SARS-CoV infection will help us understand how the innate immune system responds to and protects animals from CoV infection.

Another compelling issue is that of exactly how the SARS-CoV macrodomain suppresses innate immune gene expression. Until recently, it was thought that the primary role for the CoV macrodomain was to dephosphorylate ADR-1′′-phosphate, a by-product of tRNA splicing, to ADR (14, 19–22, 31). Hence, it was primarily referred to as an ADRP (ADPR-1′′-phosphatase). However, this intermediate has never been detected during a CoV infection, and there is no known connection between ADR-1′′-phosphate or ADR and the innate immune system. Therefore, we believe it is much more likely that the CoV macrodomain acts as a de-MAR/PARylating enzyme than as an ADRP, and future efforts will be devoted to identifying its target protein(s).

Identifying protein targets of ARTDs is a priority for researchers, and such studies will likely identify many points of cell biology that may be regulated by macrodomains (48, 49). At this time, a few ADP-ribosylated proteins involved in innate immunity have been identified. It was recently shown that ARTD14, also known as Ti-PARP, ADP-ribosylated TBK-1, which led to inhibition of the IFN response (9). ARTD12 has been shown to localize to stress granules and interact with TRIF. Furthermore, its catalytic activity enhances NF-kB-dependent gene expression and blocks both host and virus translation during alphavirus infection (8, 50). Also, ARTDs located in stress granules are able to ADP-ribosylate Argonaute proteins, which leads to blocking of RNA interference (RNAi), leading to the increased translation of ISGs (4, 5). Finally, ARTD15 was shown to ADP-ribosylate PERK and IRE1α, 2 endoplasmic reticulum (ER) stress sensors required for the induction of the unfolded-protein response (UPR) (51). ARTDs are also auto-ADP-ribosylated, which may impact their specific interactions with proteins in these pathways, making them potential macrodomain targets as well. As ARTDs are often found in stress granules, it will be of interest to determine if nsp3 localizes to these sites, in addition to replication compartments.

In addition, viral proteins may be ADP-ribosylated to modify their functions. Recently, two influenza A virus proteins, PB2 and PA, were shown to be PARylated and targeted for ubiquitin-dependent degradation when overexpressed. This degradation was countered by the coexpression of the PB1 protein of influenza A virus, explaining why this degradation does not occur during infection (52). As the SARS-CoV macrodomain was unable to block cytokine expression in isolation, it is possible that it removes antiviral ADP-ribosylation from SARS-CoV proteins. Furthermore, nsp3 is a transmembrane protein mostly localized within virus replication complexes, which may limit its access to ADP-ribosylated cellular proteins. While several viral proteins could be targeted by the macrodomain, likely targets include the PL^pro^ domain of nsp3, which is located in close proximity to the macrodomain and contains deubiquitinase activity that can repress innate immune signaling (reviewed in reference 53), and the N protein, which binds to nsp3 and is also able to block innate immune signaling in overexpression (54, 55). Experiments are under way to identify specific protein targets of the SARS-CoV macrodomain.

The nsp3 macrodomain is completely conserved across the *Coronavirinae* subfamily, suggesting that it plays a key role in the CoV lifecycle. Recent work, including this study, has clearly shown that this domain is critical for the virulence of CoVs and their ability to suppress the innate immune response (19, 20, 27). Identifying the molecular targets of the CoV macrodomains will further enhance our understanding of how CoVs evade the innate immune response and address the issue of why this domain has been conserved throughout coronavirus evolution. Resolution of such issues will improve our ability to identify new strategies and targets for antiviral therapies.

## MATERIALS AND METHODS

### Cell culture, plasmids, and reagents.

Vero E6, Huh-7, 293T, and HeLa cells were grown in Dulbecco’s modified Eagle medium (DMEM) supplemented with 10% fetal bovine serum (FBS), and Calu-3 2B4 cells (Kent Tseng, University of Texas Medical Branch) were grown in MEM supplemented with 20% FBS as previously described (41). Codon-optimized sMacro (nucleotides 3262 to 3783 of SARS-CoV MA15) and MERS-CoV ORF4a were synthesized and cloned into pUC57 (GenScript). The sMacro and GFP sequences were PCR amplified and ligated into a linearized pcDNA3 plasmid using In-Fusion (Invitrogen) cloning. The ORF4a sequence was PCR amplified and then restriction digested and ligated into the pLKO plasmid. The resulting constructs were confirmed by restriction digestion, PCR, and direct sequencing. Human IFN-α (B/D) and IFN-β were purchased from PBL (Piscataway, NJ). High-molecular-weight poly(I-C) and poly(dA-dT) were purchased from InvivoGen (San Diego, CA). Cells were transfected with either Polyjet (Amgen, Thousand Oaks, CA) or Lipofectamine 2000 (Fisher Scientific, Waltham, MA) per the instructions of the manufacturers.

### Mice.

Pathogen-free BALB/c mice were purchased from the National Cancer Institute (Frederick, MD) or Jackson Laboratories (Bar Harbor, ME). IFNAR^−/−^ mice on a BALB/c background were obtained from Joan Durbin (Rutgers-New Jersey Medical School). Mice were bred and maintained in the animal care facility at the University of Iowa. Animal studies were approved by the University of Iowa Institutional Animal Care and Use Committee (IACUC) and met stipulations of the *Guide for the Care and Use of Laboratory Animals*.

### Generation of recombinant pBAC-MA15 constructs.

All recombinant pBAC-MA15 constructs were created using Red recombination (see primers in Table S1 in the supplemental material). The recombinant BAC with the N1040A mutation (AA3382-3383GC) was engineered using a GalK/kanamycin dual marker cassette that was previously described (34, 56). Additional BACs with point mutations in the nsp3 macrodomain were engineered using the Kan^r^-I-SceI marker cassette for dual positive and negative selection as previously described (27, 33). Final BAC DNA constructs were analyzed by restriction enzyme digestion, PCR, and direct sequencing for isolation of correct clones.

### Reconstitution of recombinant pBAC-MA15-derived virus.

All work with MA15 virus was conducted in the University of Iowa biosafety level 3 (BSL3) Laboratory Core Facility. Approximately 10^6^ Vero E6 cells were transfected with 1 μg of pBAC-MA15 DNA using Lipofectamine 2000 (Fisher Scientific) as a transfection reagent. Two separate bacterial clones were used for the N1040A mutation. Viral plaques were evident by 72 to 96 h after transfection. Then, recombinant virus underwent 2 rounds of plaque purification followed by 2 amplification steps prior to its use. The resulting BAC-derived recombinant viruses used in this study are listed in Table S1.

### Virus infection.

Vero-E6 or Calu-3 2B4 cells were infected at the indicated MOIs. Infected cells and supernatants were collected, and titers were determined on Vero E6 cells. Mice were lightly anesthetized using isoflurane and were intranasally infected with 3 × 10^4^ PFU in 50 μl DMEM. To obtain tissue for virus titers, mice were euthanized at different days postchallenge, lungs were removed and homogenized in phosphate-buffered saline (PBS), and titers were determined on Vero E6 cells. Virus titers are represented as numbers of PFU/lung. Two separate clones of the N1040A virus were used in independent experiments.

### Immunoblotting.

Total cell extracts were lysed in sample buffer containing SDS, protease and phosphatase inhibitors (Roche, Basel, Switzerland), β-mercaptoethanol, and a universal nuclease (Fisher Scientific). Proteins were resolved on an SDS polyacrylamide gel, transferred to a polyvinylidene difluoride (PVDF) membrane, hybridized with a primary antibody, reacted with an infrared (IR) dye-conjugated secondary antibody, visualized using a Li-COR Odyssey Imager (Li-COR, Lincoln, NE), and analyzed using Image Studio software. Primary antibodies used for immunoblotting included anti-N polyclonal antibody (IMG548; Imgenex, San Diego, CA); anti-SARS nsp3 polyclonal antibody (kindly provided by Mark Denison, Vanderbilt University, Nashville, TN); and anti-actin monoclonal antibody (clone AC15; Abcam, Inc., Cambridge, MA). Secondary IR antibodies were purchased from Li-COR.

### Immunofluorescence.

To analyze intracellular protein localization by immunofluorescence, cells grown on glass coverslips were fixed in 4% paraformaldehyde, permeabilized with 0.1% Triton X-100 for 15 min, blocked with 1% goat serum–PBS, incubated with anti-SARS nsp3 polyclonal antibody in blocking buffer, and subsequently labeled with secondary antibody. Labeled cells were counterstained with TO-PRO-3 (Fisher Scientific) to visualize the nuclei and then mounted on slides with Vectashield antifade reagent (Vector Laboratories, Burlingame, CA). Images were captured using a Leica STED SP8 confocal laser scanning microscope, and images were analyzed using LAS X software.

### Lung cell preparation.

Lung cells were prepared as previously described (37).

### Flow cytometry.

For surface staining, cells derived from the lungs were treated with Fc block (CD16/32, 2.4G2) and then incubated with specific MAbs or isotype controls. The monoclonal antibodies used for these studies, CD45-PECy7/FITC (30-F11), CD11b-Percp-Cy5.5 (M1/70), Ly6C-APC (AL-21), Ly6G-FITC (1A8), F4/80-PE (BM8), CD11c-eFluor 450 (N418), PerCp-Cy5.5-anti-IA/IE (M5/114.15.2), CD3-APC (145-2C11), CD4-e450 (RM4-5), CD8-FITC (53-6.7), and NKP46-PECy7 (29A1.4), were purchased from BD Biosciences or eBiosciences. Cells were analyzed using a fluorescence-activated cell sorter (FACS) Verse flow cytometer (BD Biosciences, Mountain View, CA). All flow cytometry data were analyzed using FlowJo software (Tree Star, Inc., Ashland, OR).

### Real-time quantitative PCR (RT-qPCR) analysis.

RNA was isolated from tissue culture cells or from perfused lung homogenates using Trizol (Fisher Scientific) and treated with RNase-free DNase (Promega, Madison, WI), and cDNA was prepared using Moloney murine leukemia virus (MMLV) reverse transcriptase per the manufacturer’s instructions (Invitrogen). RT-qPCR was performed using an Applied Biosystems 7300 real-time PCR system (Applied Biosystems, Foster City, CA) and RT^2^ 2× SYBR green qPCR master mix (Qiagen). Primers used for qPCR are listed in Table S2. Cycle threshold (*C*_*T*_) values were normalized to those of the housekeeping gene encoding hypoxanthine phosphoribosyltransferase (HPRT) by the following equation: Δ*C*_*T*_ = *C*_T(gene of interest)_ − *C*_T(HPRT)_. All results are shown as a ratio to HPRT calculated as −2^Δ*CT*^.

### Lung histology and immunohistochemistry.

Lungs were removed, fixed in zinc formalin, and paraffin embedded. Sections were stained with hematoxylin and eosin and examined by light microscopy. For immunohistochemical staining of tissues, 10% formalin-fixed, paraffin-embedded lung sections (6 to 7 μm in thickness) were microwaved in 10 mM citrate buffer (pH 6.0) for 5 min. Endogenous peroxidase was inactivated with 3% hydrogen peroxide (H_2_O_2_) at room temperature for 10 min. Sections were then incubated (overnight at 4°C) with rabbit anti-N protein (IMG548; Imgenex, San Diego, CA) (1:1,000). Secondary labeling with biotinylated goat anti-rabbit IgG (1:200) was performed at room temperature for 1 h, followed by color development with 3,3′-diaminobenzidine for 3 min.

### Protein expression and purification.

SARS-CoV macrodomains were expressed and purified as described previously, with minor adjustments (57). Briefly, C41 (DE3) cells carrying pET vector encoding SARS-CoV macrodomain or point mutants were grown in TB media to an optical density at 600 nm (OD_600_) of ~0.6, and the cultures were cooled to 16°C and induced with 50 µM IPTG (isopropyl-β-d-thiogalactopyranoside) for 20 h. Harvested pellets were stored at −80°C until purification. The pellets were lysed with 1× BugBuster reagent–50 mM Tris (pH 7.6)–150 mM NaCl–15 mM imidazole supplemented with lysozyme, Benzonase, and phenylmethylsulfonyl fluoride (PMSF). The lysates were clarified by centrifugation, and the supernatants were subjected to nickel-nitrilotriacetic acid (Ni-NTA) resin. Proteins were eluted using 50 mM Tris (pH 7.6)–150 mM NaCl–300 mM imidazole, dialyzed to 25 mM Tris (pH 7.6)–150 mM NaCl–1 mM dithiothreitol (DTT), frozen in liquid nitrogen, and stored at −80°C.

### ARTD10 de-ADP-ribosylation assay.

ARTD10 de-ADP-ribosylation assays were performed as described previously (16, 23). Briefly, glutathione *S*-transferase (GST)-ARTD10 catalytic domain (cd) was bound to glutathione Sepharose beads in reaction buffer (50 mM HEPES [pH 7.2], 150 mM NaCl, 0.2 mM DTT, 0.02% NP-40). A 15-μl volume of bead slurry per reaction was used with 7.5 µl of ~0.5 mg/ml GST-ARTD10cd. The beads were washed with reaction buffer, and an automodification reaction was performed at 37°C for 20 min with 5 to 10 kBq ^32^P-labeled NAD^+^ per reaction, at a final NAD^+^ concentration of 1 µM. The beads were washed with reaction buffer and incubated with 0.5 µM SARS-CoV macrodomains at 37°C for 30 min. Reactions were separated by SDS-PAGE, subjected to Coomassie staining, dried, and exposed to autoradiography films. The films were quantified using ImageJ.

### Statistics.

A Student’s *t* test was used to analyze differences in mean values between groups. All results are expressed as means ± standard errors of the means (SEM). Differences in survival were calculated using a Kaplan-Meier log-rank test. *P* values of ≤0.05 were considered statistically significant (*, *P* < 0.05; ** *P* < 0.01; *** *P* < 0.001; n.s., not significant).

## SUPPLEMENTAL MATERIAL

Figure S1  SARS-CoV macrodomain mutants do not significantly affect expression or localization of nsp3. (A) Vero cells were infected with the indicated WT and mutated viruses at an MOI of 0.1 PFU/cell and collected at 24 hpi. Lysates were analyzed by immunoblotting with the indicated antibodies using a Li-COR Odyssey Imager. Nsp3 abundance was quantified by dividing the signal of the nsp3 protein by the actin signal and normalizing the ratio in WT-infected cells to 1. (B) Calu-3 2B4 cells seeded on coverslips were infected with the indicated WT viruses at an MOI of 1 PFU/cell, fixed at 48 hpi, and analyzed by immunofluorescence with anti-SARS-CoV nsp3 antibody (green) and counterstained with TOPRO-3 (red) to visualize the nuclei of the cells. Scale bars (10 μm) are shown. Download Figure S1, PDF file, 0.5 MB

Figure S2  SARS-CoV macrodomain enzymatic activity is not required for IFN resistance *in vitro*. (A to C) Vero cells were pretreated with the indicated amounts of IFN-α (A) and IFN-β (B and C) for 18 h and then infected with the indicated virus at an MOI of 0.01 PFU/cell. Cells were also subjected to posttreatment with IFN. Progeny virus was collected at 48 hpi, and titers (A and B) and genomic RNA levels (C) were determined by plaque assay and RT-qPCR with primers specific for nsp12 and normalized to HPRT, respectively. Data are derived from results from a single experiment, representative of 2 independent experiments. Download Figure S2, PDF file, 0.1 MB

Figure S3  IFN-I is not responsible for reduced N1040A virus load in lungs. IFNAR^−/−^ BALB/c mice were infected with WT and N1040A virus as described above, and lung titers at 24 hpi were determined by plaque assay. WT, *n* = 6 mice; N1040A, *n* = 7 mice. *, *P* ≤ 0.05; **, *P* ≤ 0.01; ***, *P* ≤ 0.001. Download Figure S3, PDF file, 0.03 MB

Figure S4  N1040A infection modestly affects immune cell infiltration. BALB/c mice were infected as described above, lungs were harvested at the indicated times postinfection, and the percentages and total numbers of infiltrating inflammatory cells were determined by flow cytometry. Data are derived from results of 1 experiment representative of 2 independent experiments performed with 4 mice/group/experiment. *, *P* ≤ 0.05; **, *P* ≤ 0.01; ***, *P* ≤ 0.001. Download Figure S4, PDF file, 0.3 MB

Table S1  Primers used to create recombinant BACs. Table S1, PDF file, 0.1 MB

Table S2  Quantitative real-time qPCR primers. Table S2, PDF file, 0.05 MB
