# Supplementary figures and images for: The Conserved Coronavirus Macrodomain Promotes Virulence and Suppresses the Innate Immune Response during Severe Acute Respiratory Syndrome Coronavirus Infection
Source: mBio. 2016 Dec 13;7(6):e01721-16. doi: 10.1128/mBio.01721-16 (PMC5156301; doi:10.1128/mBio.01721-16)

**A**

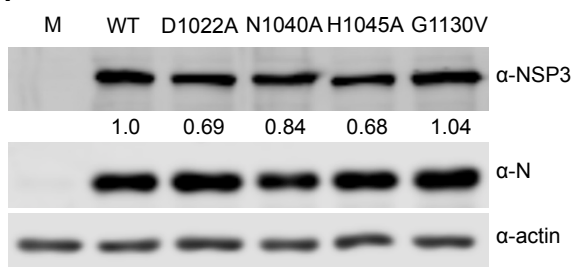

**B**

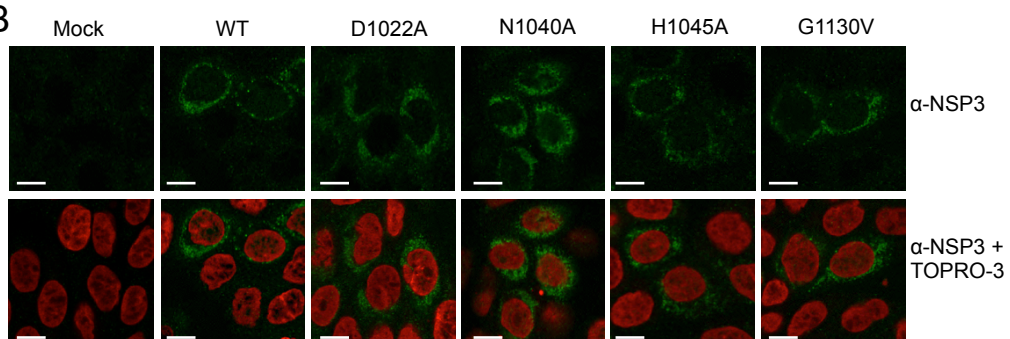

Supplement: Figure S1 — SARS-CoV macrodomain mutants do not significantly affect expression or localization of nsp3. (A) Vero cells were infected with the indicated WT and mutated viruses at an MOI of 0.1 PFU/cell and collected at 24 hpi. Lysates were analyzed by immunoblotting with the indicated antibodies using a Li-COR Odyssey Imager. Nsp3 abundance was quantified by dividing the signal of the nsp3 protein by the actin signal and normalizing the ratio in WT-infected cells to 1. (B) Calu-3 2B4 cells seeded on coverslips were infected with the indicated WT viruses at an MOI of 1 PFU/cell, fixed at 48 hpi, and analyzed by immunofluorescence with anti-SARS-CoV nsp3 antibody (green) and counterstained with TOPRO-3 (red) to visualize the nuclei of the cells. Scale bars (10 μm) are shown. Download [file mbo006163112sf1.pdf]

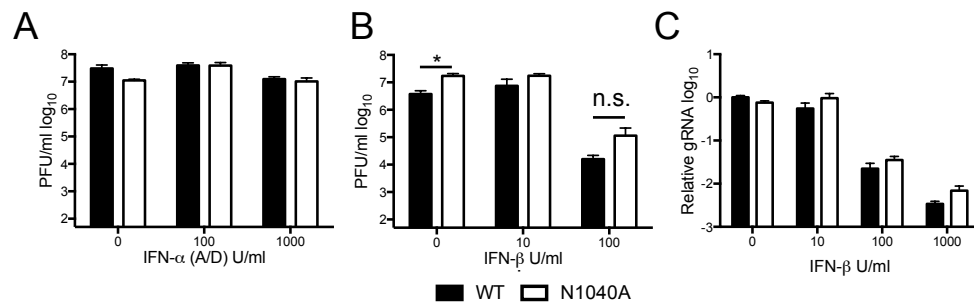

Supplement: Figure S2 — SARS-CoV macrodomain enzymatic activity is not required for IFN resistance in vitro. (A to C) Vero cells were pretreated with the indicated amounts of IFN-α (A) and IFN-β (B and C) for 18 h and then infected with the indicated virus at an MOI of 0.01 PFU/cell. Cells were also subjected to posttreatment with IFN. Progeny virus was collected at 48 hpi, and titers (A and B) and genomic RNA levels (C) were determined by plaque assay and RT-qPCR with primers specific for nsp12 and normalized to HPRT, respectively. Data are derived from results from a single experiment, representative of 2 independent experiments. Download [file mbo006163112sf2.pdf]

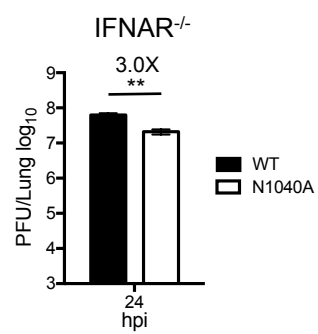

Supplement: Figure S3 — IFN-I is not responsible for reduced N1040A virus load in lungs. IFNAR−/− BALB/c mice were infected with WT and N1040A virus as described above, and lung titers at 24 hpi were determined by plaque assay. WT, n = 6 mice; N1040A, n = 7 mice. *, P ≤ 0.05; **, P ≤ 0.01; ***, P ≤ 0.001. Download [file mbo006163112sf3.pdf]

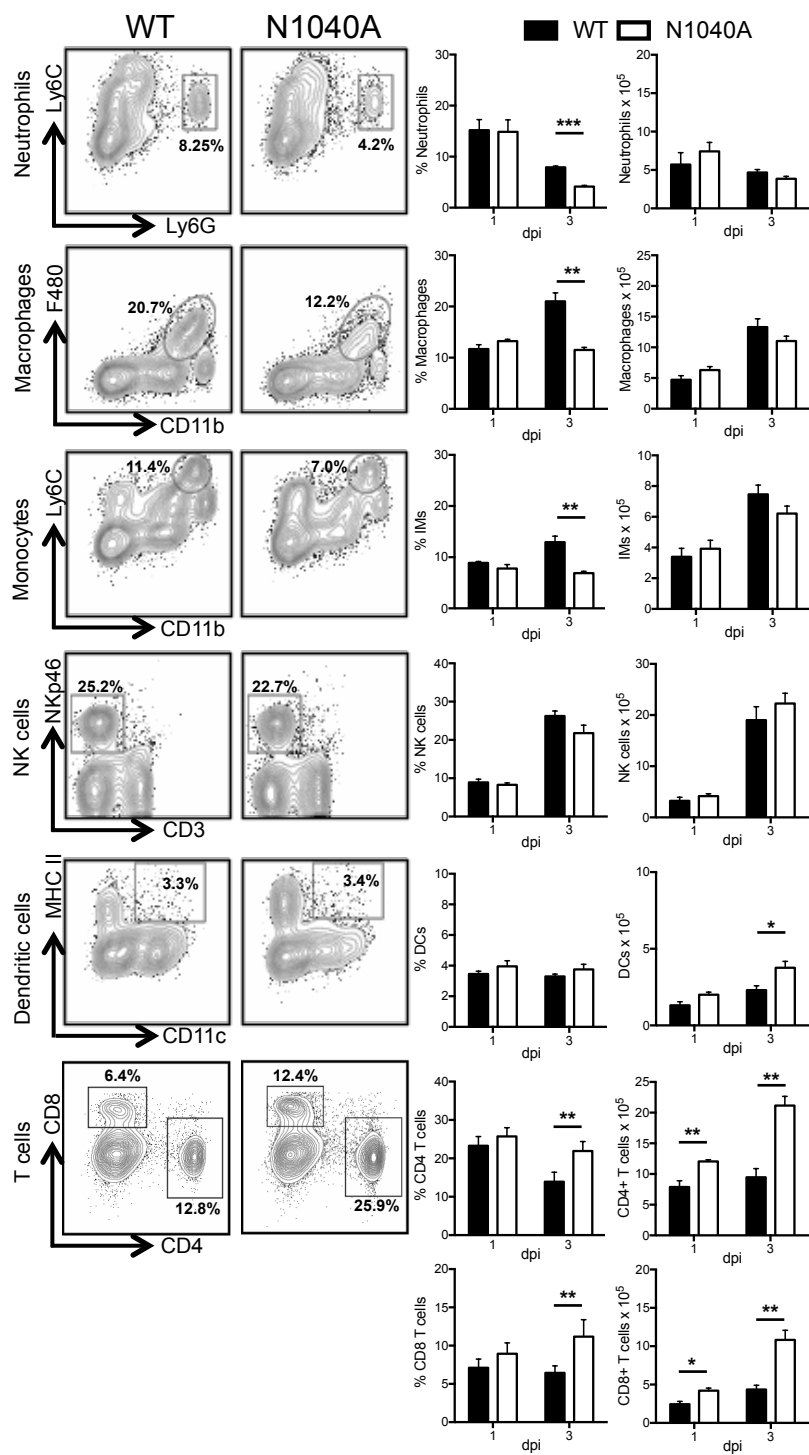

Supplement: Figure S4 — N1040A infection modestly affects immune cell infiltration. BALB/c mice were infected as described above, lungs were harvested at the indicated times postinfection, and the percentages and total numbers of infiltrating inflammatory cells were determined by flow cytometry. Data are derived from results of 1 experiment representative of 2 independent experiments performed with 4 mice/group/experiment. *, P ≤ 0.05; **, P ≤ 0.01; ***, P ≤ 0.001. Download [file mbo006163112sf4.pdf]
