# Supplementary material for: The Conserved Coronavirus Macrodomain Promotes Virulence and Suppresses the Innate Immune Response during Severe Acute Respiratory Syndrome Coronavirus Infection
Source: mBio. 2016 Dec 13;7(6):e01721-16. doi: 10.1128/mBio.01721-16 (PMC5156301; doi:10.1128/mBio.01721-16)
Supplement: Table S1 — Primers used to create recombinant BACs. [file mbo006163112st1.pdf]

Supplemental Information

Table S1. Primers used to create recombinant BACs.

Primer	Sequence	Resulting Construct
Forward primer to amplify N1040A-Galk-Kan^{R}	ATGATACTACTGAGCAATCAGAGATTGAGCCAGAACCAGAACCTACACCTcctgttgacaattaatcatcg	pBAC-MA15-Galk-Kan^{R}*sub*Macro
Reverse primer to amplify N1040A-Galk-Kan^{R}	GAATCTTCTGTGTTTGGTGGCTCCTCTTGTTTAGGTGCTTCCACTCTAGGctcagcaaaagttcgattta	
Internal forward primer to make N1040A mutation	TAAATGCTGCTGCCATACACCTG	SARS-Macro N1040A
Internal reverse primer to make N1040A mutation	CAGGTGTATGGCAGCAGCATTTA	
Forward primer to amplify SARS-Macro N1040A and insert into pBAC-MA15-nsp3-Galk-Kan^{R}	ATGATACTACTGAGCAATCAGAGATTGAGCCAGAACCAGAACCTACACCTGAAGAACCAGTTAATCAGTTT	pBAC-MA15-N1040A
Reverse primer to amplify SARS-Macro N1040A and insert into pBAC-MA15-nsp3-Galk-Kan^{R}	GAATCTTCTGTGTTTGGTGGCTCCTCTTGTTTAGGTGCTTCCACTCTAGGCTTCAGGTTATCAAGATAATC	
Forward primer to amplify H1045A-Kan^{R}-I-SceI	CCTATGGTGATTGTAAATGCTGCTAACATACACCTGAAAGCTGGTGGTGGTGTAGCAGGT aggatgacgacgataagtaggg	pBAC-MA15-H1045A
Reverse primer to amplify H1045A-Kan^{R}-I-SceI	TTGGTTGCCTTGTTGAGTGCACCTGCTACACCACCACCAGCTTTCAGGTGTATGTTAGCAcaaccaattaaccaattctgattag	
Forward primer to amplify D1022A-Kan^{R}-I-SceI	TTATTTAAAACTTACTGACAATGTTGCCATTAAATGTGTTGCCATCGTTAAGGAGGCACAaggatgacgacgataagtaggg	pBAC-MA15-D1022A
Reverse primer to amplify D1022A-Kan^{R}-I-SceI	TCACCATAGGATTAGCACTTTGTGCCTCCTTAACGATGGCAACACATTTAATGGCAACATgccagtgttacaaccaattaacc	
Forward primer to amplify L1127A-Kan^{R}-I-SceI	ATATGAAAATTTCAATTCACAGGACATCTTACTTGCACCAGCGTTGTCAGCAGGCATATTaggatgacgacgataagtaggg	pBAC-MA15-L1127A
Reverse primer to amplify L1127A-Kan^{R}-I-SceI	CTGAAGTGGTTTAGCACCAAATATGCCTGCTGACGCCAATGGTGCAAGTAAGATGTCCTGgccagtgttacaaccaattaacc	
Forward primer to amplify G1130V-Kan^{R}-I-SceI	CAATTCACAGGACATCTTACTTGCACCATTGTTGTCAGCAGTGATATTTGGTGCTAAACCaggatgacgacgataagtaggg	pBAC-MA15-G1130V
Reverse primer to amplify G1130V-Kan^{R}-I-SceI	ACACTTGTAAAGACTGAAGTGGTTTAGCACCAAATATCACTGCTGACAACAATGGTGCAAgccagtgttacaaccaattaacc	

Mutations depicted in Red

Virus specific sequence in uppercase

Marker specific sequence in lowercase
