# Supplementary material for: The Conserved Coronavirus Macrodomain Promotes Virulence and Suppresses the Innate Immune Response during Severe Acute Respiratory Syndrome Coronavirus Infection
Source: mBio. 2016 Dec 13;7(6):e01721-16. doi: 10.1128/mBio.01721-16 (PMC5156301; doi:10.1128/mBio.01721-16)
Supplement: Table S2 — Quantitative real-time qPCR primers. [file mbo006163112st2.pdf]

Table S2. Quantitative real-time qPCR primers.

Gene	Primers	Gene	Primers
mHPRT	5'-gcgtcgtgattagcgatgatg-3'	hHPRT	5'-tgaggatttggaaagggtg-3'
	5'-ctcgagcaagtctttcagtcc-3'		5'-gcacacagagggctacaatg-3'
mIFN-β	5'-tcagaatgagtggtggttgc-3'	hIFN-β	5'-gtctcctccaaattgctctc-3'
	5'-gacctttcaaatgcagtagattca-3'		5'-acaggagcttctgacactga-3'
mCXCL-10	5'-gccgtcattttctgcctcat-3'	hCXCL-10	5'-cgattctgatttgctgccttat-3'
	5'-gcttccctatggccctcatt-3'		5'-ggcttgcaggaataatttcaagt-3'
mIL-6	5'-gctaccaaactggatataatcagga-3'	hIL-6	5'-attctgcgcagctttaagga-3'
	5'-ccaggtagctatggtactccagaa-3'		5'gaggtgcccatgctacattt-3'
mTNF-α	5'-gaactggcagaagaggcact-3'	hTNF-α	5'cctgggattcaggaatgtgt-3'
	5'-agggtctgggccatagaact-3'		5'aggccccagtttgaattctt-3'
mISG-15	5'-ggccacagcaacatctatga-3'	SARS nsp12	5'-agggagtttgaccttgttcag-3'
	5'-cgcaaatgcttgatcactgt-3'		5'-ataatgcacctgtcatcctcg-3'
mIFN-α4	5'-tccatcagcagctcaatgac-3'		
	5'-aggaagagagagggctctccag-3'		
